# Genetic Analysis and Fine Mapping of a Spontaneously Mutated Male Sterility Gene in *Brassica rapa* ssp. *chinensis*

**DOI:** 10.1534/g3.120.401091

**Published:** 2020-02-11

**Authors:** Tzu-Kai Lin, Ya-Ping Lin, Shun-Fu Lin

**Affiliations:** * Department of Agronomy, National Taiwan University, Taipei, Taiwan, R.O.C. 10617; ‡ Institute of Ecology and Evolutionary Biology, National Taiwan University, Taipei, Taiwan, R.O.C.; † Crop Science Division, Taiwan Agricultural Research Institute, Taichung, Taiwan, R.O.C. 41362

**Keywords:** Non-heading Chinese cabbage, Genic male sterility, Mutant, Genotyping by sequencing (GBS), SNP, Plant breeding

## Abstract

Male sterility has been widely used in hybrid seed production in Brassica, but not in *B. rapa* ssp. *chinensis*, and genetic models of male sterility for this subspecies are unclear. We discovered a spontaneous mutant in *B. rapa* ssp. *chinensis*. A series of progeny tests indicated that male sterility in *B. rapa* ssp. *chinensis* follows a three-allele model with *BrMs^a^*, *BrMs^b^*, and *BrMs^c^*. The male sterility locus has been mapped to chromosome A07 in BC_1_ and F_2_ populations through genotyping by sequencing. Fine mapping in a total of 1,590 F_2_ plants narrowed the male sterility gene *BrMs* to a 400 kb region, with two SNP markers only 0.3 cM from the gene. Comparative gene mapping shows that the *Ms* gene in *B. rapa* ssp. *pekinensis* is different from the *BrMs* gene of *B. rapa* ssp. *chinensis*, despite that both genes are located on chromosome A07. Interestingly, the DNA sequence orthologous to a male sterile gene in *Brassica napus*, *BnRf*, is within 400 kb of the *BrMs* locus. The *BnRf* orthologs of *B. rapa* ssp. *chinensis* were sequenced, and one KASP marker (BrMs_indel) was developed for genotyping based on a 14 bp indel at intron 4. Cosegregation of male sterility and BrMs_indel genotypes in the F_2_ population indicated that *BnRf* from *B. napus* and *BrMs* from *B. rapa* are likely to be orthologs. The BrMs_indel marker developed in this study will be useful in marker-assisted selection for the male sterility trait.

Male sterility is often used in plant breeding to facilitate the production of hybrid seeds. It can be classified into cytoplasmic male sterility (CMS), which relies on cytoplasmic organelle genes; and genic male sterility (GMS), which is determined in the nucleus. Although CMS was widely applied in plant breeding, it was associated with undesirable agricultural traits such as poor growth, decreased seed production and reduced disease resistance (Levings 1990). Thus, GMS has become an alternative approach to overcome the disadvantages of CMS. Many spontaneous GMS mutants have been discovered in Brassica, including in cauliflower (Nieuwhof 1961), cabbage (Fang *et al.* 1997), non-heading Chinese cabbage (Cao and Li 1981; Ying *et al.* 2003), rapeseed (Pan *et al.* 1988), Chinese cabbage (Takahata *et al.* 1996; Feng *et al.* 2009) and turnip (Kenji *et al.* 2013).

The need to produce large quantities of hybrid seed limits the commercial application of GMS for two reasons. First, when a gene controlling GMS is recessive, *msms* for male sterility for example, half of the progenies in a male sterile line will restore fertility when male sterile plants are crossed to male fertile plants (*Msms*) during seed propagation of the male sterile line. Those male fertile plants in the male sterile line must be eliminated during hybrid seed production, increasing labor costs. Second, when GMS is controlled by a dominant gene (van der Meer 1987), male sterile plants have genotype *Msms*. The male sterile plant has no pollen so it cannot self-pollinate to obtain *MsMs* inbred lines. Dominant male sterility is commercially feasible when genes controlling GMS are either multi-allelic (Song *et al.* 2006; Feng *et al.* 2009) or involve interaction of multiple genes (Li *et al.* 1985; Chen *et al.* 1998; Lu *et al.* 2004). Under these genetic models, dominant male sterile homozygotes are obtained through sib-mating within male sterile lines. Crossing dominant GMS plants with recessive temporary maintainer lines can produce all male sterile progenies as female parents for hybrid seed production.

Numerous DNA markers closely linked with GMS genes have been developed for marker-assisted breeding and have contributed to positional cloning (Ying *et al.* 2003; Ke *et al.* 2004; Lu *et al.* 2004; Ke *et al.* 2005; Hong *et al.* 2006; Song *et al.* 2006; He *et al.* 2008; Hong *et al.* 2008; Feng *et al.* 2009; Xu *et al.* 2009). A total of five GMS genes were mapped on linkage groups N7, N16, N19 and A8 in *B. napus* (Yi *et al.* 2006; Huang *et al.* 2007; Lei *et al.* 2007; Xiao *et al.* 2008; Lu *et al.* 2013). Two GMS genes were mapped on linkage groups A8 and R7, respectively, in Chinese cabbage (*B. rapa* ssp. *pekinensis*), (Feng *et al.* 2009; Zhang *et al.* 2010), while only one recessive GMS gene was mapped on linkage group A2 in non-heading Chinese cabbage (*B. rapa* ssp. *chinensis*) (Li *et al.* 2016). Although many gene mapping studies have located genetic regions conferring GMS, few proved allelism of the GMS genes (Zu *et al.* 2010; Xie *et al.* 2012). Moreover, comparative mapping of GMS genes from various genetic materials remains difficult due to insufficient marker density and lack of common polymorphic markers with known physical positions.

Construction of reduced-representation libraries (RRL) followed by next-generation sequencing is a convenient method to obtain large numbers of DNA markers to characterize genetic variation. Genotyping by sequencing (GBS) allows multiplexing large numbers of individuals at low cost in an efficient manner (Elshire *et al.* 2011). GBS is particularly useful for generating high density single nucleotide polymorphism (SNP)-based genetic maps with physical positions revealed by alignment to a reference genome. GBS-based QTL mapping has been applied to many crop species including but not limited to corn, wheat, barley, cabbage and melon (Elshire *et al.* 2011; Poland *et al.* 2012; Lee *et al.* 2015; Chang *et al.* 2017).

We found a male sterile individual originating from a spontaneous mutation in landraces of non-heading Chinese cabbage. The male sterile line derived from this mutant has a commercial advantage because it is able to produce crossbred progenies entirely with male sterility for hybrid seed production. This new GMS mutant provides an important genetic resource since few studies have reported the location of GMS and in fact, only one recessive GMS gene was discovered previously (Cao and Li 1981; Ying *et al.* 2003; Li *et al.* 2016). Moreover, comparing this new GMS mutation in *B. rapa* ssp. *chinensis* with close relatives is interesting, since *B*. *rapa* ssp. *pekinensis* might be derived from natural crosses between *B*. *rapa* ssp. *chinensis* and *B*. *rapa* ssp. *rapifera* (Li 1981); and *B. rapa* (2n = 20, genome AA) is one of the progenitors of *B. napus* (2n = 38, genome AACC). This study focuses on determining the inheritance of GMS, constructing genetic maps to locate the GMS locus using GBS, developing SNP markers closely linked to GMS for marker-assisted selection, and clarifying the evolution of GMS genes in the genus Brassica.

## Materials And Methods

### Plant materials

**Figure 1:**
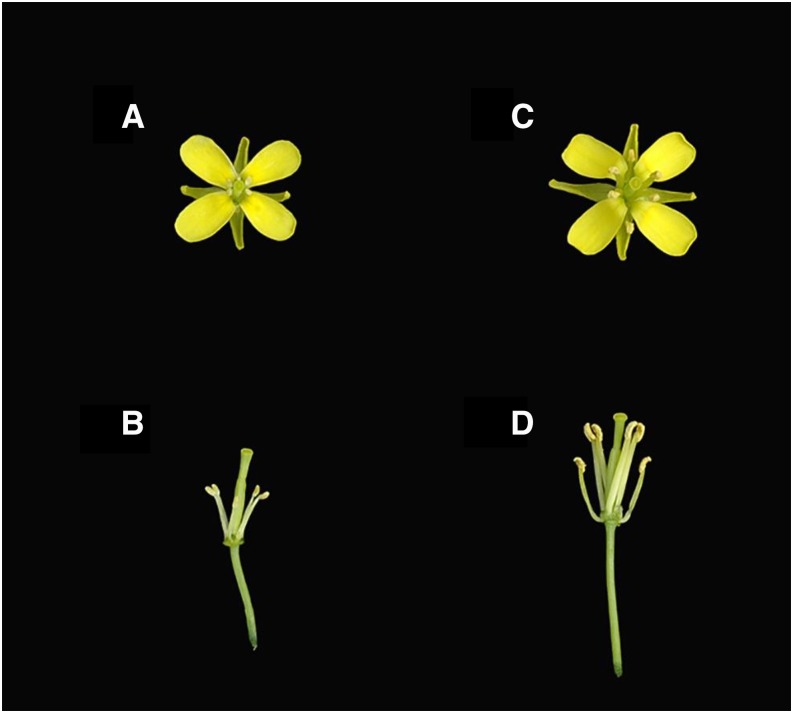
Floral organ morphology of male sterility: A and B are NH80-A (male sterile); C and D are NH80-B (male fertile).

A spontaneous mutant of GMS was discovered from landrace NH80 of non-heading Chinese cabbage (*B. rapa* ssp. *chinensis* var. *oleifera* Makino), which is used as green manure in Taiwan. NH80-AB was a GMS line developed by sib mating of male sterile and male fertile NH80 individuals for eight generations. A cross of male sterile (NH80-A) with male fertile (NH80-B) plants would result in half of the progenies being sterile. Morphologically, the male sterile plants display shrunken and pale anthers with no fertile pollen and shortened filaments, clearly distinguishable from the male fertile plants (Figure 1). The restorer line, TA95, and maintainer line, WH606, were *B. rapa* ssp. *chinensis* inbred lines respectively derived from germplasm collected in Thailand and Taiwan, and developed by at least eight generations of selfing.

### Genetic analysis of male sterility

Genetic analysis of spontaneous male sterility in this study used Liu’s (1992) progeny test method with minor modifications. There are two genetic male sterility models considered: one is a ‘multi-allelic’ model determined by one gene with multiple alleles, the other is a ‘two-gene’ model determined by two dominant genes. Under the first model, the male sterility gene, *BrMs*, has a total of three alleles: *BrMs^a^*, *BrMs^b^* and *BrMs^c^*, with *BrMs^b^* conferring GMS and dominant to *BrMs^c^*. If this model is true, then *BrMs^b^Ms^b^* and *BrMs^b^Ms^c^* would be sterile while *BrMs^a^* could restore fertility by suppressing *BrMs^b^*. Under the second model, *BrMs* represents the GMS gene while *BrRf* is a different gene recovering male fertility. Individuals with the genotypes *BrMsMs rfrf* and *BrMsms rfrf* would be male sterile because *BrRf* will suppress *BrMs* to restore fertility.

Three single cross combinations, NH80-A × TA95, NH80-A × WH606, and TA95 × WH606, were completed by intercrossing single plants from male sterile line NH80-A, restorer TA95 and maintainer WH606 (Table1). Progeny tests A, B and C were conducted to examine male sterility at flowering time. Progeny test A, including 64 F_2_ subpopulations of NH80-A × TA95, was used to test segregation of GMS in at least 60 individuals for each F_2_ subpopulation. Progeny test B was used to test the segregation of GMS in hybrids from crosses between 40 randomly chosen male sterile F_2_ individuals from progeny test A and WH606, the maintainer line. At least 20 progeny per cross were investigated for male sterility. Progeny test C was used to test allelism of GMS by crossing the restorer (TA95) with the maintainer line (WH606), examining sixty individual plants from each of 25 F_2_ subpopulations for segregation of male sterility. The genotypes of each line under the two proposed genetic models are listed in Table S1. A total of eight possible genotypic combinations by crossing male sterile individuals with restorer lines, and their theoretical segregation ratios, are shown in Tables S2-S4.

**Table 1:** Sterility of progenies in different crosses between male sterile, restorer, and maintainer lines

Cross	No. of male sterile plants	No. of male fertile plants
NH80-A × TA95	0	70
NH80-A × WH606	40	0
TA95 × WH606	0	50
TA95⊗*^a^*	0	72
WH606⊗*^a^*	0	70

a ⊗ indicates self-pollination.

### Mapping populations

A total of 88 F_2_ plants derived from NH80-A × TA95 and 186 BC_1_ plants derived from NH80-A × WH606 were used for mapping of *BrMs^a^/BrMs^b^* and *BrMs^b^*/*BrMs^c^*, respectively. Plants were grown in individual pots. DNA was extracted from leaves of each 30-day old plant for constructing GBS libraries. Phenotypic data were collected at flowering for each plant.

### Genotyping by sequencing

GBS libraries were constructed based on Poland *et al.* (2012) with some modifications. Approximately 200 ng DNA of each individual was digested with restriction enzymes *Pst*I-HF and *Msp*I (New England Biolabs [NEB], Ipswich MA, US) at 37° for 4 h and then kept at 65° for 20 min. The digested DNA was ligated with the barcoded and reverse Y-adapters (Poland *et al.* 2012), in a total volume of 40 μL containing 200 ng of digested DNA, 20 nM barcoded adapter, 300 nM reverse Y-adapter, 200 U of T4 DNA ligase, 1 × NEB Buffer 4 (NEB, Ipswich, MA, USA), and 1 mM ribo-ATP at 22° for 2 h and then at 65° for 20 min. Two DNA libraries were established by mixing 15 μL of the above samples from each individual of F_2_ and BC_1_ populations, respectively. The DNA libraries were purified using AMPure XP beads at the ratio of 1:1.8 for DNA:beads (Beckman Coulter, Brea CA, USA); modified with Illumina primers (Poland *et al.* 2012); and amplified for 18 cycles at 95° for 30 s, at 62° for 25 s, and at 68° for 40 s. These two GBS libraries were sequenced with Illumina Hiseq2000 (100-bp, single-end; Illumina Inc., San Diego, CA, USA) in the Genome Research Center, National Yang-Ming University, Taiwan.

The reference genome *B. rapa* var. Chiifu40 version 1.5 (http://brassicadb.org/brad/index.php) was used for data analysis (Cheng *et al.* 2011). The raw reads of 100-bp single-end sequences were selected for high-quality reads (Q > 20), then sorted according to barcodes using CLC Genomics Workbench (version 8.0.2; CLC Bio, http://www.clcbio.com). Clean reads without barcode sequences were mapped onto the reference genome and saved in the .sam file format. Locus indexing, SNP calling, and genotyping of segregants were performed using the command ‘ref_map.pl’ in Stacks v. 1.37 (http://creskolab.uoregon.edu/stacks/) (Catchen *et al.* 2011). Unique sequences of clean reads were defined as tags. Only tags with depth of more than five reads in each individual and missing in less than 20% of a population were used as DNA markers for genotyping. Files S1 and S2 contain physical positions of polymorphic tags and genotypes for each individual of F_2_ and BC_1_ populations, respectively.

### Linkage analysis of BrMs

Genetic maps of the F_2_ and BC_1_ populations were constructed with the R/qtl program (Broman *et al.* 2003; Broman 2010). Linkage groups were formed with pair-wise recombination of tag markers smaller than 0.3 and LOD scores greater than 7. The Kosambi function (Kosambi 1943) was used to calculate genetic distance. Closely linked tag markers (genetic distance smaller than 0.5 cM) were merged into the same bin, with the threshold being at least one recombination event between adjacent bins. A random tag marker within each bin was selected as a bin marker. The genetic distance was recalculated with respect to the randomly selected bin markers. Male sterility was considered a morphological marker, and was added to the linkage analysis.

### Development of SNP and Indel markers linked to BrMs

Tags co-segregating with the *BrMs* gene were developed into SNP markers with Kompetitive Allele Specific PCR (KASP) assays (LGC Genomics, Teddington, UK). In addition, two SCAR markers, syau_SCR01 and syau_SCR04 (Feng *et al.* 2009), were converted into SNP markers. The two primer pairs of the SCAR markers were used to amplify DNA segments of NH80-A and TA95, and the amplicons were sequenced using an ABI Prism 3730 DNA sequencer (Applied Biosystems, Foster City, USA). BioEdit (version 7.2.0) (Hall 1999) was used for DNA sequence alignment. In addition, the DNA sequence of the *BnRf* gene (GenBank accessions No. KT818624 and No. KT818625) was referenced to design primer pairs for comparative sequencing of NH80-A and TA95 lines, and variation regions in the DNA sequences within the candidate gene were used to design Indel marker with Kompetitive Allele Specific PCR (KASP) assays.

A total of 1,590 F_2_ plants derived from NH80-A × TA95 were genotyped with SNP markers for fine-mapping. The developed Indel KASP marker was used for allelism analysis related to *BnRf* and *BrMs* genes. DNA samples of each individual plant were isolated with QuickExtract DNA extraction solution (Epicentre, Madison, WI, USA). Applied Biosystems Viia 7 Real‐Time PCR System (Applied Biosystems, Foster City, USA), was used for fluorescence detection according to the manufacturer’s protocol from KASP, and Viia 7 software (v. 1.2) was used for genotype calling.

### Data availability

All polymorphic tags and genotypic data are available in Supplementary Files S1 and S2. DNA sequences of *BnRf* orthologs in NH80-A and TA95 are available in Supplementary Files S3. Linkage maps of two populations are available in Supplementary Figures S1 and S2. The predicted genotypes of two genetic models of GMS are listed in Supplementary Table S1, as well as segregation ratios of male sterility in three sets of progeny tests are available in Supplementary Tables S2, S3, and S4. Supplemental material available at figshare: https://doi.org/10.25387/g3.11663970.

## Results

### Genetic model of GMS in Brassica rapa ssp. chinensis

Selfed progenies of TA95 and WH606, and F_1_ progenies of NH80-A × TA95, TA95 × WH606 show male fertility, while F_1_ progenies of NH80-A × WH606 are male sterile (Table 1). The GMS allele in NH80-A is homozygous and dominant to WH606. The GMS genotype of NH80-A should be *BrMs^b^Ms^b^* under the multi-allelic model described in the M&M, or *BrMsMs rfrf* under the two-gene model. The corresponding maintainer genotypes should be *BrMs^c^Ms^c^* under the multi-allelic model or *Brmsms rfrf* under the two-gene model, and the corresponding restorer genotypes should be *BrMs^a^Ms^a^* or *BrMsMs RfRf*, *BrMsms RfRf*, *Brmsms RfRf*, as shown in Table S1.

**Figure 2:**
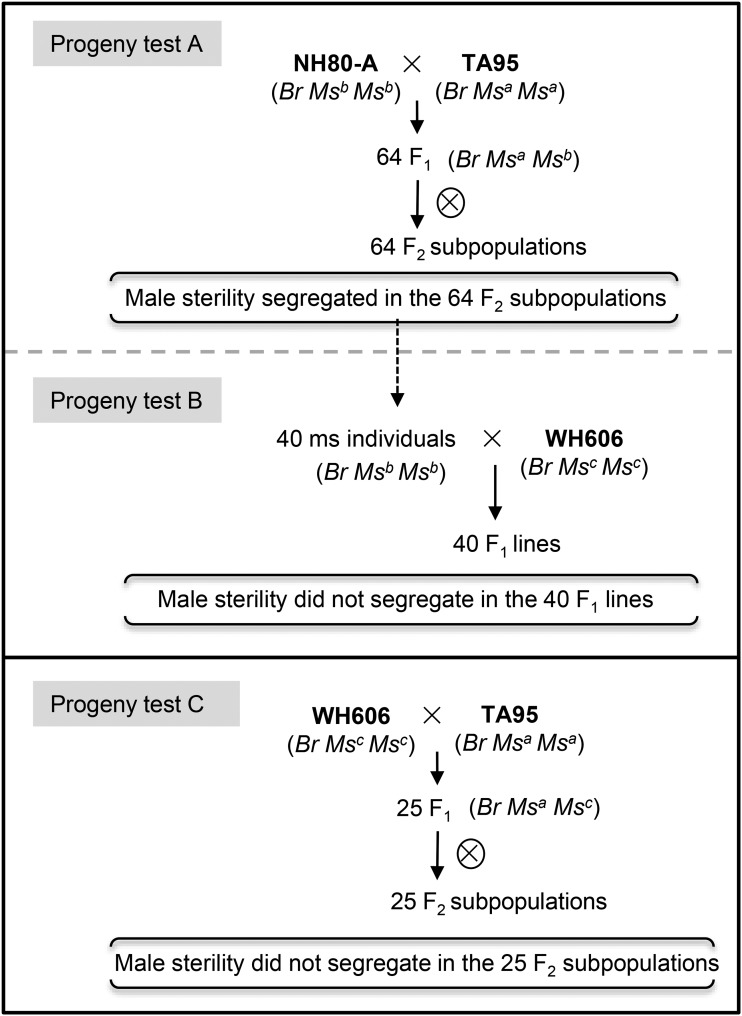
Inheritance of genetic male sterility in three different progeny tests. The genotypes of male sterile line NH80-A, restorer line TA95, maintainer line WH606, and progenies are indicated in brackets under the multiple-allele model.

In progeny test A, segregation of male sterility was observed in a total of 64 F_2_ subpopulations derived from NH80-A × TA95. In progeny test B, all plants in each of 40 F_1_ lines were male sterile (Figure 2). These two progeny tests suggest that the possible genotypes of NH80-A and TA95 are *BrMs^b^Ms^b^* and *BrMs^a^Ms^a^* respectively, under the multi-allelic model or *BrMsMs rfrf* and *BrMsMs RfRf* under the two-gene model (Table S2, Table S3). In progeny test C, all F_2_ plants derived from TA95×WH606 were fertile (Figure 2), suggesting *BrMs^a^* and *BrMs^c^* are allelic (Table S4). All three progeny tests suggest that the inheritance of GMS follows a multi-allelic model, with *BrMs^a^* dominant to *BrMs^b^*, and *BrMs^b^* dominant to *BrMs^c^* (*BrMs^a^* > *BrMs^b^* > *BrMs^c^)*. Therefore, the best fit of the model to the observed data are that the genotype of NH80-A is *BrMs^b^Ms^b^*; the genotype of NH80-B is *BrMs^a^Ms^b^*; the genotype of restorer line TA95 is *BrMs^a^Ms^a^*, and the genotype of WH606 is *BrMs^c^Ms^c^*.

### Linkage analysis of BrMs

An F_2_ population derived from NH80-A × TA95 was used to map the *BrMs* locus containing *BrMs^a^* and *BrMs^b^* alleles. A total of 25 Gb of raw data were acquired from the Illumina sequencing platform, and 245 million cleaned reads with an average length of 93.6 bp were obtained after quality trimming and mapped to the reference genome. Read mapping rates of the male sterile line NH80-A, restorer line TA95, and the F_2_ populations are 81.7%, 41.9%, and 80.2%, respectively. A total of 33,632 tags were aligned to the reference genome with 5,284 being polymorphic between the parents. After eliminating markers with more than 20% missing data, we obtained a total of 2,243 tag markers to construct the linkage map (Table 2). After assigning tags with the same genotypes into bins, the final genetic map comprises 569 recombination bins with 4,347 SNPs on ten linkage groups. The length of the genetic map is 1,011 cM, with the longest linkage group spanning 135.8 cM (chromosome A09), and the shortest spanning 75.1 cM (chromosome A10). The average distance between consecutive bins is 1.8 cM, ranging from 1.6 to 2.1 cM. The number of polymorphic tags per bin ranges from 3.5 to 5.0, with an average of 3.9.

**Table 2:** Characteristics of the linkage groups established in the F_2_ and BC_1_ populations for mapping *BrMs*

Characteristics	Chromosomes										Total
	A01	A02	A03	A04	A05	A06	A0 7	A08	A09	A10	
F_2_ population (H_o_: *BrMs^a^/BrMs^b^*)											
Length (cM)	107.8	98.2	132.6	93.4	107	102.8	82	76.5	135.8	75.1	**1,011**
No. of SNP	384	468	527	306	400	572	495	335	584	276	**4,347**
No. of polymorphic Tag	206	233	263	176	209	279	256	185	288	148	**2,243**
No. of Bin	58	60	68	44	54	62	51	49	82	41	**569**
average SNPs (SNPs/Tag)	1.9	2.0	2.0	1.7	1.9	2.1	1.9	1.8	2.0	1.9	**1.9**
average Tags (Tags/bin)	3.6	3.9	3.9	4.0	3.9	4.5	5.0	3.8	3.5	3.6	**3.9**
average interval (cM/bin)	1.9	1.6	2.0	2.1	2.0	1.7	1.6	1.6	1.7	1.8	**1.8**
BC_1_ population (H_o_: *BrMs^b^/BrMs^c^*)											
Length (cM)	108.4	116.5	135.3	81.2	99.6	108.8	99.7	79.9	142.1	79.8	**1,051**
No. of SNP	192	229	189	87	167	217	156	155	283	116	**1,791**
No. of polymorphic Tag	117	117	100	42	90	132	87	98	175	58	**1,016**
No. of Bin	55	59	55	32	38	57	43	44	77	33	**493**
average SNPs (SNPs/Tag)	1.6	2.0	1.9	2.1	1.9	1.6	1.8	1.6	1.6	2.0	**1.8**
average Tags (Tags/bin)	2.1	2.0	1.8	1.3	2.4	2.3	2.0	2.2	2.3	1.8	**2.1**
average interval (cM/bin)	2.0	2.0	2.5	2.5	2.6	1.9	2.3	1.8	1.8	2.4	**2.1**

**Figure 3:**
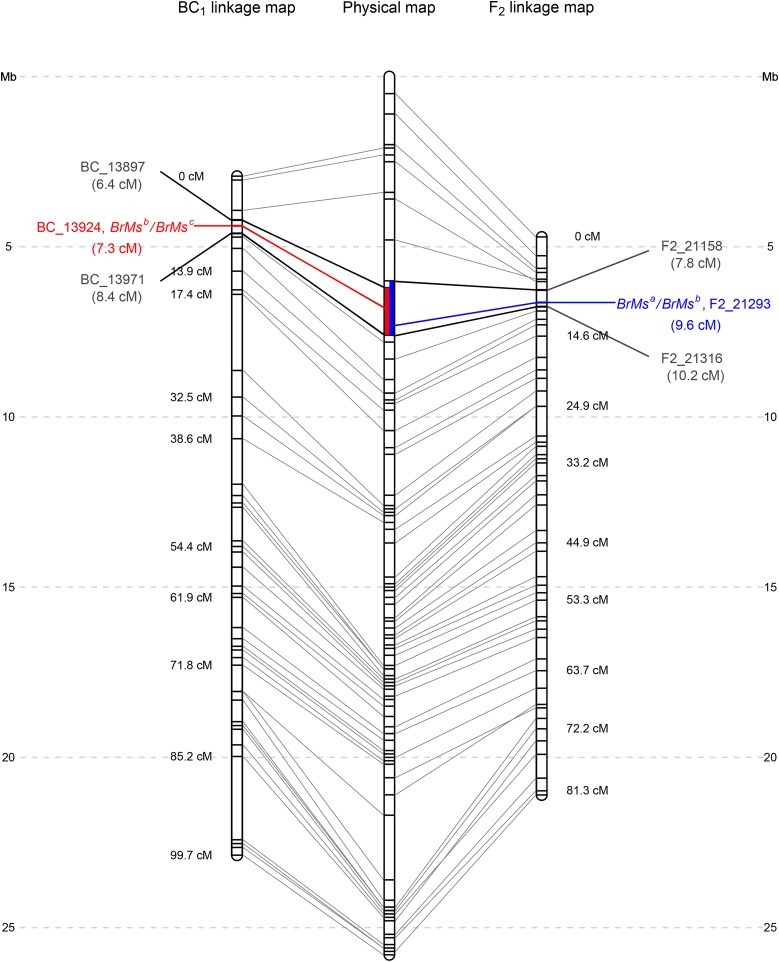
Linkage maps of the male sterility gene in non-heading Chinese cabbage. The *BrMs^b^*/*BrMs^c^* locus mapped on chromosome A07 in the BC_1_ population (left), and the *BrMs^a^/BrMs^b^* locus mapped on chromosome A07 in the F_2_ population (right). The overlapping physical maps of *BrMs^b^*/*BrMs^c^* and *BrMs^a^/BrMs^b^* are indicated by red and blue bars.

The *BrMs* locus was mapped on chromosome A07 in the F_2_ population of NH80-A × TA95 (Figure 3; Figure S1). The length of chromosome A07 is 82 cM, containing 51 bins. The bin “F2_21293” is cosegregating with *BrMs^a^*, while the two flanking bins, “F2_21158” and F2_21316”, are 1.8 and 0.6 cM from *BrMs^a^*, respectively. The corresponding physical position of *BrMs^a^* is approximately 6.0 to 7.6 Mb (Table 3, Figure 3). Within this 1.6 Mb, there are a total of four tags (F2_21230, F2_21231, F2_21236, and F2_21271) cosegregating with the *BrMs^a^* gene in addition to F2_21293, and the flanking bins, F2_21158 and F2_21316 contain 3 and 2 tags, respectively. The regions of the ten tags contain a total of 15 SNPs (Table 3). The *BrMs^a^* locus can be further narrowed to a 1.0 Mb interval within the 1.6 Mb region based on the physical locations of the 10 tags.

**Table 3:** GBS tags cosegregating with the *BrMs* gene

Bin*^a^*	Tags*^b^*	SNP numbers	Genetic map (cM)	Physical map (bp)*^c^*
F_2_ population (H_o_: *BrMs^a^/BrMs^b^*)				
**F2_21158**	F2_21158	1	7.78	6,043,218
	F2_21208	1		6,498,810
	F2_21222	1		6,632,406
F2_21293*	F2_21230	1	9.63	6,700,179
	F2_21231	2		6,700,608
	F2_21236	3		6,710,545
	F2_21271	3		7,038,629
	F2_21293	1		7,297,575
**F2_21316**	F2_21316	1	10.24	7,597,365
	F2_21322	1		7,625,231
BC_1_ population (H_o_: *BrMs^b^/BrMs^c^*)				
**BC_13879**	BC_13879	2	6.44	6,211,318
BC_13924*	BC_13914	1	7.30	6,700,179
	BC_13917	1		6,700,978
	BC_13923	1		6,736,962
	BC_13924	1		6,750,243
**BC_13971**	BC_13971	1	8.41	7,583,322

a Bold markers indicate bin markers. Bins segregated with *BrMs^a^* or *BrMs^c^* are indicated by *.

b Tags in the same bins displayed the same segregation pattern.

c The physical map of the first nucleotide of each tag is indicated. *B*. *rapa* var. Chiifu40 (v1.5) was used for reference sequences, downloaded from the Brassica database (http://brassicadb.org/brad/index.php).

The BC_1_ population derived from NH80-A × WH606 was also used to map the *BrMs* locus, containing *BrMs^b^* and *BrMs^c^* alleles. A total of 25 Gb of raw data were also acquired from the Illumina sequencing platform, and 250 million cleaned reads with an average length of 92.6 bp were obtained after quality trimming. After aligning the clean reads to the reference genome, we found that the mapping rate for the maintainer (WH606) and the BC_1_ populations were 85.4%, and 84.4%, respectively. A total of 22,224 tags were obtained in the BC_1_ population, fewer than those from the F_2_ population (33,632). After filtering, 1,016 polymorphic tags were obtained including 1,791 SNPs which were combined into 493 recombination bins. A linkage map for the BC_1_ populations comprises the 493 recombination bins spanning a total of 1,051 cM on ten chromosomes. The longest linkage group is chromosome A09, spanning 142.1 cM and the shortest is chromosome A10, spanning 79.8 cM (Table 2). The average distance between consecutive bins was 2.1 cM, while four pairs of bins have gaps larger than 10 cM (Figure S2). Bins contain an average of 2.1 polymorphic tags, ranging from 1.3 to 2.4 tags (Table 2).

In this NH80-A × WH606 BC_1_ population, the *BrMs* locus was also mapped to chromosome A07 (Figure 3; Figure S2). *BrMs^c^* was cosegregating with a total of four tags, bin BC_13924, BC_13914, BC_13917 and BC_13923 and flanked by bins BC_13879 and BC_13971 at distances of 0.9 and 1.1 cM from the *BrMs* locus. The physical position of *BrMs^c^* is in the range from 6.2 to 7.6 Mb (Table 3, Figure 3). The fact that the physical positions of *BrMs^a^* and *BrMs^c^* overlap in the range of 6.6–7.6 Mb (Figure 3) and tags BC_13914 and F2_21230 were located at the same position, essentially the same tag, indicates that *BrMs^a^*, *BrMs^b^*, and *BrMs^c^* are allelic, in concordance with the results from the three progeny tests (Figure 2).

### Fine-mapping of BrMs

**Figure 4:**
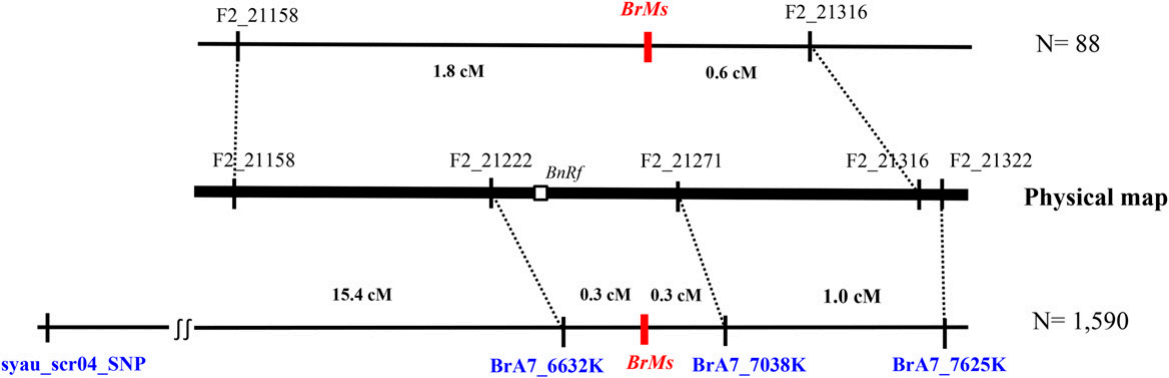
Fine mapping of the GMS gene. *BrMs* was fine mapped using 1,590 individuals in the F_2_ population of NH80-A × TA95 and SNP markers. Loci on the linkage and physical maps are illustrated. The corresponding physical positions of *BnRf* on *B*. *napus* are indicated by an open box.

SNPs cosegregating with or flanking *BrMs* within the tags were designed into SNP markers for marker-assisted selection (Table 4). A total of 1,590 individuals in the NH80-A × TA95 F_2_ population were used to fine map the *BrMs* locus. The flanking SNP markers, BrA7_6632K and BrA7_7625K, are 0.3 and 1.3 cM from the *BrMs* locus, respectively (Figure 4). Another SNP marker, BrA7_7038K, obtained from the cosegregating tags, was on the same side as BrA7_7625K, but only 0.3 cM from the *BrMs* locus. Therefore, the *BrMs* locus was finely mapped to a 0.6 cM region, corresponding to a 400 kb physical region. Only three (0.19%) of 1,590 plants were recombinants, suggesting that these two SNP markers are appropriate for marker-assisted selection.

**Table 4:** Primer sequences of SNP and Indel markers for KASP assays

SNP marker	Tag	KASP Assay	Sequence
BrA7_6632K	F2_21222	Allele 1 Primer	5′-CACCAACAACGCATCATCGTCC-3′
		Allele 2 Primer	5′-CACCAACAACGCATCATCGTCT-3′
		Common Primer	5′-GTTTGACCTAGCTCGACGAAACCAA-3′
BrA7_7038K	F2_21271	Allele 1 Primer	5′-CTGAAGCTTTGTCGATTTTGTAAGTAATA-3′
		Allele 2 Primer	5′-CTGAAGCTTTGTCGATTTTGTAAGTAATG-3′
		Common Primer	5′-CCGGCAACGCTTCCTTTACCTCTA-3′
BrA7_7625K	F2_21322	Allele 1 Primer	5′-AAGAAGTTTGATGAAGAGGTTTCTTCAATA-3′
		Allele 2 Primer	5′-GAAGTTTGATGAAGAGGTTTCTTCAATG-3′
		Common Primer	5′-CACCAAATTACCCAACCTGTTCATTTCTT-3′
syau_scr04_SNP	—	Allele 1 Primer	5′-TGAGTCTAAGATAAGCATGCATCATC-3′
		Allele 2 Primer	5′-GAGTCTAAGATAAGCATGCATCATT-3′
		Common Primer	5′-TTAACACAGGTTGCTAACAGGATATATCTT-3′
BrMs_indel	—	Allele 1 Primer	5′- CGATAAGATCCCTCTTAAGTCTTAAG -3′
		Allele 2 Primer	5′- CGATAAGATCCCTCTTAAGTCTTAAC -3′
		Common Primer	5′- CCAGTACAACAATTGTAGATACAGAGACAA -3′

Two SCAR markers, syau_SCR01 and syau_SCR04, developed from Chinese cabbage (Feng *et al.* 2009) was used to compare GMS between *B. rapa* ssp. *chinensis* and *B. rapa* ssp. *pekinensis* in the same F_2_ population. These two SCAR markers produced the same amplicons between Chinese cabbage and non-heading Chinese cabbage, with no length differences. Sequencing showed that the amplicon of the primer “syau_scr04” contained a SNP between NH80-A and TA95. This SNP was designed into a SNP marker named “syau_scr04_SNP” (Table 4). Linkage analysis of the F_2_ population suggests that syau_scr04_SNP is located on chromosome A07, 15.4 cM from the *BrMs* locus (Figure 4). This result is different from a previous study (Feng *et al.* 2009) that reported a distance of 2.5 cM between syau_SCR04 and *Ms* in *B. rapa* ssp. *pekinensis*.

The GMS gene of *B. napus*, *BnRf*, was mapped to a 13.8 kb interval of LG N7, corresponding to a 17.8 kb interval of LG A7 which contains a total of three putative genes, *Bra014989*, *Bra014990*, and *Bra014991* (Xie *et al.* 2012). These three genes are located in an interval of 6,733,932–6,752,184 bp on chromosome A07, which is within the range containing the 400 kb *BrMs* locus from this study (Figure 4).

**Figure 5:**
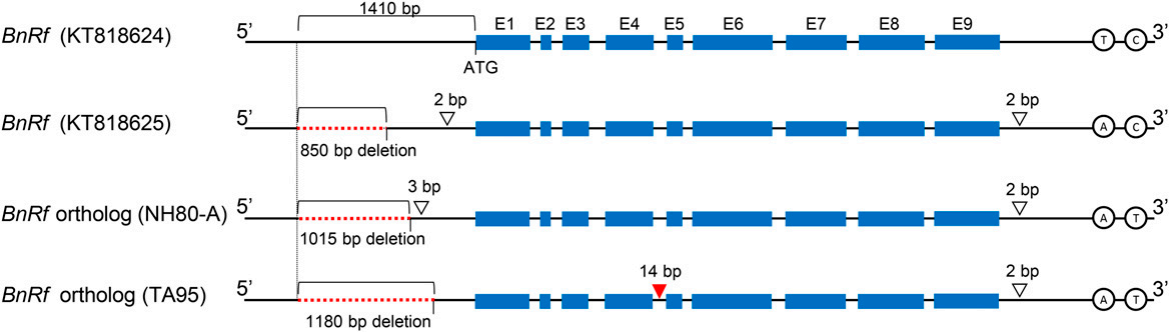
The sequence alignment of *BnRf* orthologs. The accession numbers KT818624 and KT818625 in GenBank represent the *BnRf* genes in the male sterile line 9012A and male fertile line RG206H, respectively. E1 to E9 indicate the exons of *BnRf* while the black lines indicate introns or untranslated regions. Red dotted lines indicate the deletion regions in the promoter. The colorless inverted triangles show the insertions, and inverted triangle in red displays the region of BrMs_indel marker. The circles at 3′UTR show the location of SNPs. All the labeled positions are in comparison to ATG on KT818624.

To understand the relationship between *BnRf* and *BrMs* genes, the orthologs of *BnRf* in lines NH80-A and TA95 were sequenced. The sequence length of *BnRf* orthologs in *B. napus* was longer than those *of B. rapa*. The orthologs in male sterile line 9012A and male fertile line RG206H of *B. napus* were 8,572 bp (KT818624) and 7,726 bp (KT818625), respectively (Deng *et al.* 2016). The length of orthologs in male sterile line NH80-A and in male fertile line TA95 of *B. rapa chinensis* were 7,562 bp and 7,411 bp, separately (Supplementary Files S3). Despite variation in sequence lengths, the four *BnRf* orthologs had the same DNA sequences in all 9 exons. The notable difference among the four orthologs was located at the promoter region. Compared to KT818624, the other orthologs had different lengths of deletion starting from position -1410 upstream of the translation start site (ATG). In addition to a deletion of 850 bp in *B*. *napus* male fertile line RG206H (KT818625)(Deng *et al.* 2016), 1,015 bp and 1,180 bp deletions were found in the orthologs of lines NH80-A and TA95, respectively (Figure 5). Besides, 4 insertions (2 bp and 3 bp at 5′ UTRS; 14 bp at intron 4; 2 bp at 3′ UTRS) and 2 SNP (at 3′ UTRS) were observed at non-coding regions. Based on the 14 bp insertion at intron 4, a KASP assay was conducted to investigate the indel marker (called BrMs_indel) for understanding the relationship between *BnRf* and *BrMs* (Table 4). A total of 923 F_2_ plants derived from NH80-A × TA95 were genotyped with BrMs_indel. The marker completely cosegregated with male sterility, suggesting that *BrMs* and *BnRf* genes were orthologs differentiated by mutations.

## Discussion

### A multiple allelic model of male sterility in Brassica rapa ssp. chinensis

This study first proved that the GMS in *B. rapa* ssp. *chinensis* fits a one gene multi-allelic model. A conventional method uses segregation ratios to test genetic models for GMS by conducting progeny tests in F_2_ populations derived from male sterile lines crossed with restorer lines. The theoretical ratio of male sterile and male fertile individuals in a segregating population for the multi-allele model is 1:3 while for the two-gene model is 3:13. However, it is quite difficult to separate these two ratios and draw a firm conclusion. Our study, using three progeny tests, is advantageous because we only need to determine whether there is (or is not) segregation of male sterility in the progeny populations and not distinguish subtle differences in segregation ratios. In three progeny tests, the ‘two dominant genes’ model proposed by Liu (1992) assumed that the two dominant genes were located on different chromosomes and segregated independently. However, if the two genes were linked on the same chromosome, the results of the progeny test might be biased and thus lead to a different conclusion. In our study, we used different populations to map the GMS locus in each population conferring a different allele. The result suggests that *BrMs^a^/BrMs^b^* and *BrMs^b^/BrMs^c^* map to an interval from 6.0 Mb to 7.6 Mb on chromosome A07 (Figure 3) in two different populations (F_2_ and BC_1_). Because the same tags are cosegregating with the GMS locus in both populations, it suggests that *BrMs^a^*, *BrMs^b^* and *BrMs^c^* alleles are located at the same locus.

Multiple allelism of GMS has also been validated in *Brassica rapa* ssp. *pekinensis* using two types of progeny tests and in *B. napus* using closely linked markers (Feng *et al.* 1996; Song *et al.* 2006; Liu *et al.* 2008; Xie *et al.* 2012). Our study, integrating the results of progeny tests, the same cosegregated tag in two linkage maps and the physical positions of *BrMs*, supports the multi-allelic genetic model for GMS in *B. rapa* ssp. *chinensis*. Collectively, results from *B. rapa* ssp. *pekinensis*, *B. napus* and our study, suggest that multi-allelic control of GMS in Brassica is a widespread phenomenon, consistent with the hypothesis proposed by Lu *et al.* (2013). In practice, GMS by either model can produce 100% male sterile progenies for commercialization by crossing the male sterile lines to the maintainer lines. In addition, applying GMS from a multi-allelic model with 6 possible genotypes is easier than the two dominant gene model with 9 possible genotypes.

### Construction of high-density linkage maps with GBS

We constructed high-density linkage maps of the NH80-A × TA95 F_2_ and NH80-A × WH606 BC_1_ populations, respectively, using GBS (Table 2, Figures S1–S2). Approximately twice as many SNPs were discovered in the F_2_ population as the BC_1_ population, mainly due to higher genetic diversities between the parents. A total of 81.7% and 85.4% of the Illumina reads from the parents NH80-A and WH606, originating from Taiwan, were mapped to the reference genome, while only 41.5% of the reads from TA95, originating from Thailand, were mapped. This difference in mapping rate suggests that TA95 is more genetically distant from NH80-A and WH606, corresponding to the higher frequency of polymorphic tags in the F_2_ population derived from NH80-A × TA95 (Table 2). In general, the distribution of bins in the F_2_ population is more even than that in the BC_1_ population. The two linkage maps display extensive colinearity and consistency (Figure 3).

Genetic maps of the F_2_ and BC_1_ populations are 1,101 and 1,051 cM in length, respectively, within the range of previously published linkage maps of *B. rapa* ranging from 970 cM to 1,230 cM (Suwabe *et al.* 2006; Choi *et al.* 2007; Kim *et al.* 2009; Wang *et al.* 2011; Chung *et al.* 2014; Huang *et al.* 2017). Many SNP markers from GBS are prone to genotypic errors; increased marker numbers challenge computational speed and efficiency (van Os *et al.* 2005), which inflates total genetic distances (Hackett and Broadfoot 2003). Using the ‘bin’ method to combine closely linked markers can substantially increase computational efficiency and reduce genetic distance inflation caused by genotypic errors. This method of constructing linkage groups has been widely applied to many other crops (Sun *et al.* 2007; Ganal *et al.* 2011). Benefitting from the bin approach, the total genetic distance in our study is similar to those of maps constructed using PCR-based markers (Suwabe *et al.*, 2006; Choi *et al.*, 2007; Wang *et al.*, 2011). A similar appoach was applied to construct high-density linkage maps in non-heading Chinese cabbage with restriction-site-associated DNA sequencing (RAD-seq) (Huang *et al.* 2017). The resulting genetic map contains a similar number of intervals as our F_2_ map. However, our study contains more polymorphic tags, possibly due to using different restriction enzymes or different thresholds to define tags. The previous study used the restriction enzyme *EcoR*I while our study used *Pst*I to construct GBS libraries. Flanking sequences of different restriction enzyme recognition sites may generate different sets of SNPs. Both linkage maps provide useful information for the *B. rapa* genome. Integrating these two linkage maps to obtain a relatively high-density map would facilitate investigation and applications of genomic analysis, gene mapping, and molecular breeding in *B. rapa* ssp. *chinensis*.

### Comparing the GMS mutation in Brassica rapa ssp. chinensis with those in other close relatives

The *BrMs* locus of *B. rapa* ssp. *chinensis* discovered in this study and the *Ms* locus of *B. rapa* ssp. *pekinensis* belong to the same chromosome, but the two loci appear to be different. We developed a SNP marker (syau_scr04_SNP), equivalent to the SCAR marker syau_scr04, closely linked to GMS in *B. rapa* ssp. *pekinensis* and found that it is 15.4 cM from the *BrMs* locus, while a previous study (Feng *et al.* 2009) reported a genetic distance of 2.5 cM between syau_scr04 and the *Ms* locus. Although genetic distances can vary in different populations, the long genetic distance revealed by gene fine mapping in our study suggests that *BrMs* and *Ms* are likely two different loci. Similar results have been published in previous studies. Two independent GMS loci mapped to the same LG were also reported in *B. napus*. The *BnRf* locus was mapped to LG N7 in a *B. napus* population developed from accession 9012A (Xiao *et al.* 2008; Xie *et al.* 2012), and *Bnms1* was mapped to the same LG in a different population developed from accession S45A (Yi *et al.* 2006). Nevertheless, the GMS of 9012A and S45A were not allelic (Chen *et al.* 1998), indicating that at least 2 diffferent GMS loci were on the same LG.

The *BnRf* gene from *B. napus* and *BrMs* gene from *B. rapa* are likely to be orthologs. In fact, chromosome N7 of *B. napus* and chromosome A7 of *B. rapa* are evolved from a common ancestral chromosome (http://www.brassica.info/resource/maps/lg-assignments.php); and the regions of *BnRf* on LG N7 of *B. napus* and LG A7 of *B. rapa* show high colinearity (Xie *et al.* 2012). In this study, the finding of the BrMs_indel marker cosegregating with the *BrMs* locus has demonstrated that *BnRf* and *BrMs* are allelic. Since the *BnRf* gene and the *BrMs* gene are orthologs, it is very conceivable that the *BnRf* gene of the AACC genome of *B. napus* was derived from the AA genome of *B. rapa* ssp. *chinensis*.

Long sequence insertion or deletion in promoters of the four *BnRf* orthologs is a potentially important factor affecting gene function. In *B*. *napus*, reduction in transcription efficiency may result from an 850 bp insertion in the promoter region of *BnRf* gene in male sterile line RG206A (Deng *et al.* 2016). In this study, a 165 bp insertion or deletion was also observed in the corresponding regions of NH80-A and TA95 *BnRf* orthologs. We speculated that sequence variation of this region may change male sterile gene expression in Brassica families. However, to understand the key sequences conferring variation among the four *BnRf* orthologs that affect male sterility, a further study such as genetic transformation is needed.

### Development of BrMs markers for marker-assisted selection

Three SNPs and one KASP (BrMs_indel) marker associated with male sterility of *B. rapa* ssp. *chinensis* were developed in this study. The BrMs_indel marker designed on the gene is predominantly suggested for marker-assisted selection. However, if the BrMs_indel is not available due to lack of polymorphism between breeding lines, SNP markers, BrA7_6632K and BrA7_7038K, respectively only 0.3 cM away from the *BrMs* locus are recommended (Figure 4). Either marker can assist in selecting male sterile plants in a segregating population. Using both SNP markers simultaneously will improve the selection accuracy by detecting single recombinants, only missing very rare double recombinants. These markers together with syau_scr04_SNP may facilitate selection for male sterile lines or individuals among subspecies of *B. rapa*.
